# *Pteropus lylei *primarily forages in residential areas in Kandal, Cambodia

**DOI:** 10.1002/ece3.5046

**Published:** 2019-03-13

**Authors:** Kinley Choden, Sébastien Ravon, Jonathan H. Epstein, Thavry Hoem, Neil Furey, Marie Gely, Audrey Jolivot, Vibol Hul, Chhoeuth Neung, Annelise Tran, Julien Cappelle

**Affiliations:** ^1^ Institut Pasteur du Cambodge Phnom Penh Cambodia; ^2^ EcoHealth Alliance New York New York; ^3^ Fauna & Flora International (Cambodia) Phnom Penh Cambodia; ^4^ Harrison Institute Sevenoaks UK; ^5^ CIRAD, UMR TETIS Montpellier France; ^6^ UMR TETIS, CIRAD, CNRS, IRSTEA, AgroParisTech Montpellier University Montpellier France; ^7^ CIRAD, UMR ASTRE Montpellier France; ^8^ UMR ASTRE CIRAD, INRA, Montpellier University Montpellier France; ^9^ UMR EpiA INRA Marcy l'Etoile France

**Keywords:** distribution model, ecology, epidemiology, flying fox, GPS, habitat use, interface, Nipah virus, telemetry

## Abstract

1. Bats are the second most species‐rich Mammalian order and provide a wide range of ecologically important and economically significant ecosystem services. Nipah virus is a zoonotic emerging infectious disease for which pteropodid bats have been identified as a natural reservoir. In Cambodia, Nipah virus circulation has been reported in *Pteropus lylei*, but little is known about the spatial distribution of the species and the associated implications for conservation and public health.
2. We deployed Global Positioning System (GPS) collars on 14 *P. lylei *to study their movements and foraging behavior in Cambodia in 2016. All of the flying foxes were captured from the same roost, and GPS locations were collected for 1 month. The habitats used by each bat were characterized through ground‐truthing, and a spatial distribution model was developed of foraging sites.
3. A total of 13,643 valid locations were collected during the study. Our study bats flew approximately 20 km from the roost each night to forage. The maximum distance traveled per night ranged from 6.88–105 km and averaged 28.3 km. Six of the 14 bats visited another roost for at least one night during the study, including one roost located 105 km away.
4. Most foraging locations were in residential areas (53.7%) followed by plantations (26.6%). Our spatial distribution model confirmed that residential areas were the preferred foraging habitat for *P. lylei*, although our results should be interpreted with caution due to the limited number of individuals studied.
5. *Synthesis and applications*: Our findings suggest that the use of residential and agricultural habitats by *P. lylei* may create opportunities for bats to interact with humans and livestock. They also suggest the importance of anthropogenic habitats for conservation of this vulnerable and ecologically important group in Cambodia. Our mapping of the probability of occurrence of foraging sites will help identification of areas where public awareness should be promoted regarding the ecosystem services provided by flying foxes and potential for disease transmission through indirect contact.

## 1 INTRODUCTION

Bats are the second most species‐rich Mammalian order with over 1,300 species worldwide (Voigt & Kingston, 2016) and provide a wide range of ecologically important and economically significant ecosystem services (Kunz, Torrez, Bauer, Lobova, & Fleming, 2011). They are also recognized as reservoir hosts for highly pathogenic viruses such as Nipah virus (NiV; Calisher, Childs, Field, Holmes, & Schountz, 2006).

Nipah virus was first identified in pigs and people in Malaysia in 1998 (Chua, 2000) and has reemerged annually in Bangladesh since 2001 (Luby et al., 2009). NiV causes lethal encephalitis in people, and bats in the *Pteropus* genus are the reservoir (Epstein, Field, Luby, Pulliam, & Daszak, 2006). Transmission of the virus in Malaysia is presumed to have occurred as a result of pigs consuming bat‐contaminated fruits, followed by contamination of humans working with pigs (Chua, 2003). In Bangladesh, direct bat‐to‐human transmission of the virus occurs through the consumption of date palm sap (Luby et al., 2006). NiV has been isolated or detected in several *Pteropus *species in Southeast Asia, including *P. medius* in Bangladesh, *P. lylei* in Thailand and Cambodia, and *P. vampyrus* and *P. hypomelanus* in Malaysia. However, despite its detection in *P. hypomelanus*, a serological study on Tioman Island did not find the virus in any of the local people (Chong, Tan, Goh, Lam, & Bing, 2003) that the bats live among and regularly interact with (Aziz, Clements, Giam, Forget, & Campos‐Arceiz, 2017). Seasonal NiV shedding patterns have been suggested for *P. lylei* in Thailand, with peak shedding occurring in May (Cappelle, Hul, Duong, Tarantola, & Buchy, 2014; Wacharapluesadee et al., 2010).

Understanding the capacity of a reservoir to spread the virus at local and regional levels to humans and domestic animals is fundamental to surveillance and prevention initiatives. Knowledge about the distribution and movement patterns of these bat species is thus required, and telemetry (measurement and transmission of data from remote sources) is a valuable tool to monitor the drivers and characteristics of fruit bat movements (Smith et al., 2011). This can be used to develop appropriate host management strategies that maximize the conservation of bat populations and minimize the risk of disease outbreaks in domestic animals and humans.

Telemetry studies have been undertaken on several *Pteropus* species in Asia and Australia. In Australia, tracking of fourteen *P. poliocephalus* males revealed that these are highly mobile between roosts and regularly travel long distances (Roberts, Catterall, Eby, & Kanowski, 2012). For instance, one *P. alecto* was tracked between Papua New Guinea and Australia and traveled more than 3,000 km over 11 months (Breed, Field, Smith, Edmonston, & Meers, 2010). In Southeast Asia, the movements of seven *P. vampyrus* males encompassed Malaysia, Indonesia, and Thailand, indicating the need for regional management plans for such species (Epstein et al., 2009). These studies highlight the difference between migratory and nomadic flying foxes and the need to adapt management strategies to relevant geographic scales.

At a local scale, telemetry studies indicate that *Pteropus* bats make foraging flights on a nightly basis, with distances from the roost ranging from a few kilometers to 20–30 km. Depending on species, foraging sites range from apparently intact forest to cultivated areas. In Bangladesh, the roosting ecology of *P. giganteus* is associated with forest fragmentation, likely because fragmented forests offers more foraging options to the bats, including fruit species cultivated by humans (Hahn et al., 2014). Conversely, in the Philippines, most foraging locations of eight *Acerodon jubatus* were situated in closed forest remote from areas of evident human activity (de Jong et al., 2013). Another study on *A. jubatus* and *P. vampyrus* in the Philippines suggested these species prefer undisturbed forest types and select against disturbed and agricultural areas (Mildenstein, Stier, Nuevo‐Diego, & Mills, 2005). Foraging also repeatedly occurred 15–30 km from the roost on average. Similarly, movements of *P. alecto *were very similar between nights with most foraging sites located less than 6 km from roost sites. In Thailand, *P. lylei* also undertakes relatively short foraging movements (2.2–23.6 km) on a nightly basis to fields, plantations, backyards, and mangroves (Weber et al., 2015).

In Cambodia, three flying fox species are thought to occur, large flying fox *P. vampyrus *which is listed as “near threatened” by IUCN, Lyle's flying fox *P. lylei *which is listed as “vulnerable,” and island flying fox *P. hypomelanus*, which is listed as “least concern” (IUCN, 2008; Kingsada et al., 2011). Most of the known flying fox roost sites in Cambodia are located on the grounds of pagodas, where hunting is limited by the presence of the monks (Ravon, Furey, Hul, & Cappelle, 2014). Consequently, these are often located in the middle of villages close to human and domestic animal populations, and available foraging areas mostly comprise anthropogenic landscapes. Flying foxes in Cambodia are likely to interact frequently with humans and to depend on human activities for their subsistence. As a consequence, understanding of their preferred foraging areas is important to inform public health and conservation actions.

The objective of our study was to use telemetry data to determine and characterize foraging locations visited by flying foxes inhabiting a *P. lylei* roost in Koh Thom District, Kandal Province, Cambodia.

## 2 MATERIALS AND METHODS

### 2.1 Study site

**Figure 1:**
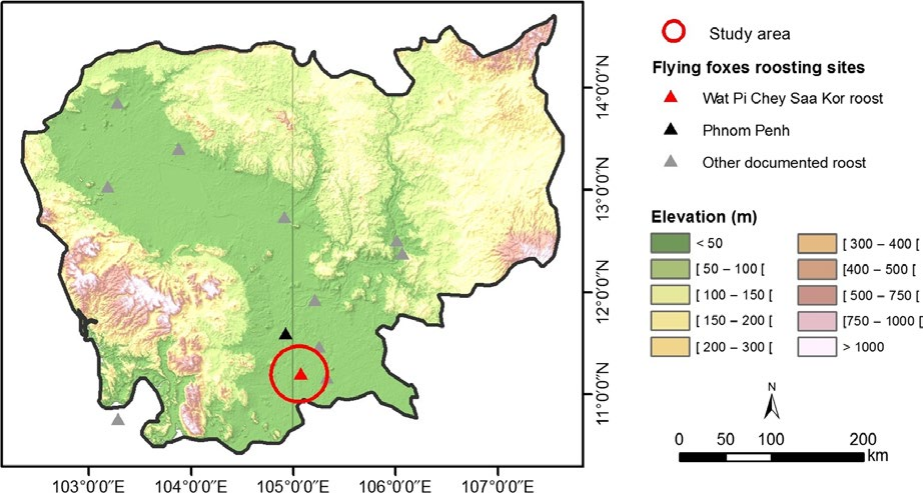
Location of the study area and other flying fox roost sites known in Cambodia

The *P. lylei *roost selected for this study was located at Wat Pi Chey Saa Kor (11.200 N, 105.058 E), Kom Poung Kor village, Koh Thom District, Kandal Province (Figure 1). The site comprises a grove of trees on the grounds of a Buddhist pagoda which encompasses 21 roost trees with an estimated population of 4,000 flying foxes (Ravon et al., 2014). The village is bisected by a road with houses on either side and is characterized by a mosaic of agriculture that lacks significant areas of natural vegetation/forest. Land uses in the region include cultivation of rice and other crops, backyards, plantations, and various backyard animal farming activities.

### 2.2 Study period

Our study was conducted from April 18, 2016 to May 17, 2016 when shedding of the NiV by *P. lylei* is believed to peak in Cambodia (Cappelle et al., 2014), similar to Thailand (Wacharapluesadee et al., 2010). Nine Global Positioning System (GPS) collars were deployed from April 18, 2016 to April 21, 2016 and five GPS collars from May 3, 2016 to May 6, 2016. Data were collected from these every day for two weeks after each collaring, related to the lifespan of individual collars.

### 2.3 Bat collaring

Bats were captured using mist nets between 6 p.m. and 5 a.m. using methods described in (Newman, Field, Epstein, & De Jong, 2011). Weight, forearm length, sex, age, and reproductive status were documented for each bat. Animals were selected for collaring based on weight. Adult males and females without pups weighing at least 400 g were selected so that collars, weighting 20 g, would comprise <5% of body mass (Brigham, 1988). Pregnant and lactating female bats were excluded to avoid adding extra burdens.

**Figure 2:**
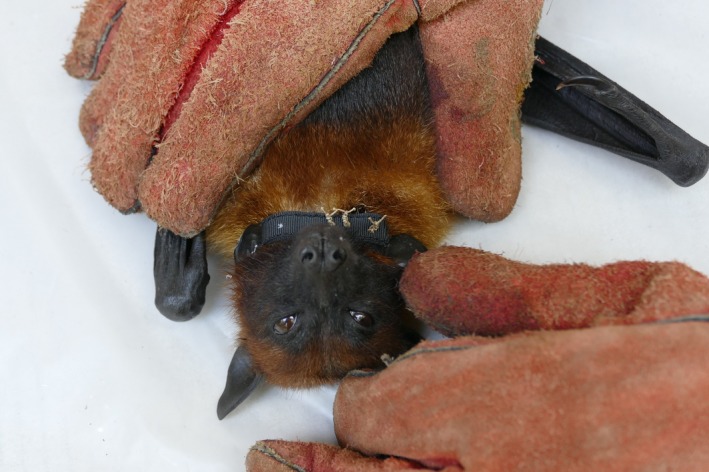
Collared *Pteropus lylei*, southern Cambodia

Selected bats were anesthetized by injecting medetomidine into the pectoral muscle (Epstein, Zambriski, Rostal, Heard, & Daszak, 2011). GPS devices (FLR V, Telemetry Solutions^™^, www.telemetrysolutions.com) attached to nylon bands were secured around the neck of each bat using catgut suture (1.0) and three surgical knots (Figure 2), which were presumed to last for at least 30 days. Following collar attachment, atipamezol was injected intramuscularly. Each collared bat was kept in a separate cage during recovery from anesthesia and offered pieces of mango ad libitum prior to release.

We deployed 14 GPS collars on 13 adult males and one adult female (Table 1). Collars 1–5 were programed to transmit one location every 30 min from 5 p.m. to 6 a.m. while collars 6–14 were programed to transmit one location every 30 min for the first night only and one location every 5 min from 5 p.m. to 6 a.m. on following nights. As a consequence, collars 1–5 were expected to last for 1 month and allow observations of foraging behavior across the expected annual excretion peak of NiV. Collars 6–14 were expected to last for 10 days and provide detailed information on *P. lylei* foraging sites, including night roosts. Data were remotely downloaded each morning from active collars with a base station, which automatically connected to the GPS collars when within reading distance (10–20 m).

**Table 1:** Characteristics of *Pteropus lylei* studied and GPS device performance, southern Cambodia. The proportion of valid data corresponds to the proportion of locations recorded with valid geographic coordinates

Bat ID	Sex	Reproductive Status	Weight (g)	Forearm (mm)	Collar lifespan (nights)	Total recorded data	Proportion of valid data (%)
Bat01	Male	Mature	560	169	26	760	32
Bat02	Male	Mature	565	152.9	3	247	90
Bat03	Male	Mature	540	165.5	11	439	81
Bat04	Male	Mature	435	NA	9	394	40
Bat05	Male	Mature	490	149.4	23	716	88
Bat06	Male	Mature	430	151.9	13	1,904	95
Bat07	Male	Mature	425	149.5	9	1,747	41
Bat08	Male	Mature	420	144.9	12	1,675	95
Bat09	Male	Mature	532	145.9	1	22	41
Bat10	Male	Mature	425	144.5	8	1,200	89
Bat11	Male	Mature	590	153.7	13	1,768	98
Bat12	Male	Mature	414	148.3	12	1,752	99
Bat13	Female	Adult	430	149.4	12	1,592	96
Bat14	Male	Mature	550	152.4	13	1,912	97

### 2.4 Spatial data and site characterization

Global Positioning System data were transferred each morning to a computer, converted into KML format (QGIS, version 2.14), and mapped to identify foraging locations visited by bats the previous night (Google Earth, version 7.1). Foraging sites were identified based on clusters of two or more locations obtained from individual bats and as many as possible were visited depending on accessibility. Tree species visited by bats and evidence of foraging such as partially eaten fruits were recorded to facilitate identification of roosting and feeding trees. Nonfruiting trees were also recorded.

### 2.5 Habitat use

All locations were classified in three major categories: roost locations (all points less than 30 m from the roost site), foraging locations (a cluster of ≥2 two points separated by <30 m where the bat spent at least 10 min at night (i.e., from 6 p.m. to 6 a.m.)), and commuting locations (isolated points connecting the roost and foraging sites located >30 m from a foraging or roost location). Based on patterns visible in Google Earth, five habitat types were recognized for foraging locations: plantations (including fruit trees within the plantation and trees around the plantation), residential areas (locations within 50 m of human settlements, including pagodas, backyards, roads), agricultural lands (any cultivated land not included in “plantations” and “residential areas”), rivers, and uncultivated areas (all locations not included in the preceding categories).

### 2.6 Spatial analysis

The home range of an animal illustrates spatial and temporal use of an area and is defined as the area commonly used for normal activities such as foraging for food, breeding, and caring for young (Burt, 1943). We used the kernelUD() function of the Adehabitat package in R software (Version 3.2.3) to estimate the home range for all bats, using the kernel density method (Calenge, 2006). The function computes the different percentage levels of home range estimation, for example the 50% home range identifies the areas where an individual is likely to occur 50% of the time.

We used QGIS to analyze the trajectories of each bat and to generate heatmaps based on kernel density estimation. The density was calculated based on the number of points in a location, with larger numbers of clustered points resulting in larger values. We also used the sp package in R software to calculate the maximum linear distance traveled from the roost per night.

The spatial distribution of foraging sites in the study area was modeled using the GPS data collected, a set of generated background data and land cover data. We created a map which classified habitats according to their expected influence on foraging site selection by the bats. This map was the product of a classification procedure based on Landsat images (30 m spatial resolution) acquired in 2015 and ground‐truthing. Details of the classification are provided as Appendix (Supporting information Appendix S1: Table S1), and the result is illustrated by (Supporting information Appendix S1: Figure S1). The eight different habitats identified in this classification were speculated to have the following impacts on the distribution of foraging sites. Plantations were expected to be highly attractive to bats because of the high density of fruit available. Tree vegetation was expected to be attractive because of the potential presence of fruit consumed by bats. Water bodies such as rivers were also expected to attract the bats due to the presence of fruit trees on their banks. Residential areas were expected to have mixed effects as a source of disturbance for the bats and a potential source of fruit in backyards. The four remaining habitats in the classification (rice field, bare soil, flooded vegetation, and shrubland) were not expected to attract the bats.

To train and validate the model, we used all GPS locations of foraging sites and generated an equivalent number of background locations in the study area which were used as pseudoabsences by the model. Half of the data were randomly assigned to a training dataset and the other half to a validation dataset. We used a generalized linear model with the training dataset as the response variable with a binomial distribution (1 for presence and 0 for pseudoabsence) and habitat type as an explanatory qualitative variable. To deal with the discrepancy between the spatial resolution of our classification (30 m) and GPS points (1–5 m), we calculated the distances of all data points to the closest habitats with an expected influence on bat habitat selection: plantations, tree vegetation, water bodies, and residential areas. Because of this discrepancy and landscape fragmentation in the study area, GPS locations of bats foraging in attractive habitats could be recorded in an adjacent nonattractive habitat. We therefore generated four explanatory variables (dPlant, dTree, dWater, and dResid) to allow us to capture the spatial structure of the study area. Using the distance to these attractive habitats as explanatory variables in the model would then help take into account the limited spatial resolution of our habitat classification as well as spatial autocorrelation. As a consequence, no further variable was added to the model to deal with the latter. Finally, distance to the roost (dRoost) was added to the explanatory variables as this should be minimized by the bats to optimize their energy efficiency while foraging. We used the results of the model, which was based on data from 14 individuals, to map the probability of presence of the foraging sites of *P. lylei* in the study area. The validation dataset was used to estimate the performance of the model through the calculation of the area under the ROC curve (AUC).

## 3 RESULTS

### 3.1 Collar performance

A total of 84 bats were caught, 14 of which were selected for collaring (Table 1). Our GPS devices transmitted from 1 to 26 nights, with an average of 11.8 nights (Table 1). A total of 13,646 valid locations were collected over 27 nights from the 14 collared bats. The proportion of valid data (i.e., data with an actual geographic location provided) varied from 32% to 99% of the data provided by each collar. Overall, 84.6% of the data generated were valid locations (*n* = 13,646/16,128).

### 3.2 Habitat use

**Figure 3:**
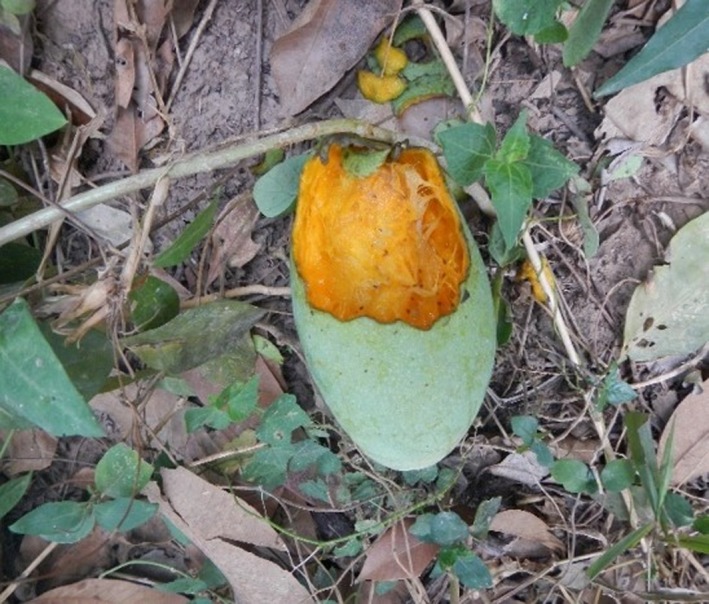
Partially consumed mangoes at a GPS foraging location of *Pteropus lylei, *Kandal Province, southern Cambodia

Tree species identified during visits to foraging sites are listed in Table 2. Partially eaten mango (*Mangifera indica, n* = 15) and sapodilla (*Manilkara zapota*, *n* = 3) were found at exact GPS foraging locations (Figure 3). It was not possible to detect whether leaves or flowers were also consumed.

**Table 2:** Tree species identified at foraging sites of 14 GPS‐collared *Pteropus lylei,* southern Cambodia

Common name	Scientific name	Species at GPS locations (5 m precision)	Species ≤30 m from GPS locations	Known to be consumed by flying foxes^a^
Banana	*Musa paradisiaca*		X	Direct
Banyan	*Ficus benghalensis*		X	Unknown
Custard apple	*Annona reticulate*		X	Direct
Eucalyptus	*Eucalyptus exserta*	X	X	Indirect
Jack fruit	*Artocarpus heterophyllus*		X	Direct
Java apple	*Syzgium malaccense*		X	Unknown
Kapok	*Ceiba pentandra*	X	X	Direct
Longan	*Dimocarpus longan*		X	Indirect
Mango	*Mangifera indica*	X	X	Direct
Neem	*Azadirachta indica*	X	X	Direct
Papaya	*Carica papaya*		X	Direct
Sacred fig	*Ficus religiosa*	X	X	Direct
Sapodilla	*Manikara zapota*	X	X	Direct
Sugar palm tree	*Borassus flabellifer*	X	X	Indirect

a Direct means direct evidence from feces or feeding remains, indirect means information based on evidence from location data but with no direct evidence from feces or feeding remains. Based on (Aziz, Clements, Peng et al., 2017; Hahn et al., 2014; Weber et al., 2015; Win & Mya, 2015).

Among the valid data, 29% of the locations (*n* = 3,959/13,646) corresponded to the roost site where the bats were captured, 20.3% (*n* = 2,774) to commuting locations, and 50.7% (*n* = 6,913) to foraging locations and night roosts. Most of the foraging locations were in residential areas: 53.7% (*n* = 3,714/6,913), 26.6% (*n* = 1,836) in plantations, 16.2% (*n* = 1,118) in uncultivated areas, 3.2% (*n* = 219) in agricultural lands, and 0.4% (*n* = 26) on rivers (Table 3). (Supporting information Appendix S1: Figure S2) shows the spatial distribution of the foraging sites in the study area.

**Table 3:** Maximum distances traveled per night by *Pteropus lylei* and proportion of foraging areas per category, southern Cambodia

Bat ID	No. of foraging locations and night roosts	Max distance/night (km)	Residential area (%)	Plantation area (%)	Agricultural land area (%)	Uncultivated area (%)	River (%)
Bat01	111	8.95	32	41	17	0	11
Bat02	145	7.91	15	75	10	0	0
Bat03	189	10.28	99	1	0	0	0
Bat04	100	29.60	75	9	16	0	0
Bat05	190	29.35	89	4	0	7	0
Bat06	1,109	23.35	32	31	4	32	1
Bat07	411	27.39	50	2	4	44	0
Bat08	798	105.14	62	17	2	19	0
Bat09	3	6.88	0	100	0	0	0
Bat10	628	52.11	18	60	2	21	0
Bat11	761	10.39	4	76	0	20	0
Bat12	964	50.33	79	8	4	9	0
Bat13	421	25.45	62	29	4	4	2
Bat14	1,083	9.03	93	2	2	2	0
Total	6,913	28.3^a^	54^b^	27^b^	3^b^	16^b^	0^b^

a mean of the maximal distance per night for all bats.

b Proportion of foraging area for all locations of all bats.

### 3.3 Movement patterns and flight distances

**Figure 4:**
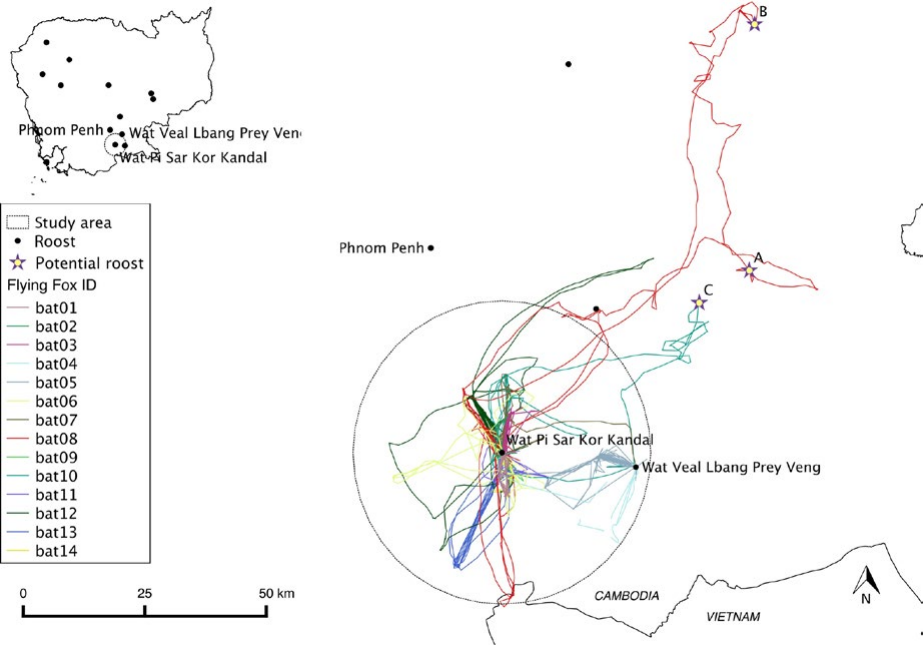
Movements of 14 GPS‐collared *Pteropus lylei* during the study period in southern Cambodia

The maximum distance traveled per bat/night ranged from 6.88 to 105.14 km and averaged 28.3 km (Table 3). All individuals showed fidelity to at least one foraging site, returning on 3–11 nights to the same site (all locations <30 m from the previous one were counted as the same foraging site) during the study period. Thirty‐six foraging sites were shared by at least two bats. All bats (excluding bat #9 due to lack of data) shared at least one and as many as eight foraging locations with another bat. Shared foraging locations or night roosts were relatively close to the roost, with an average and maximum distance of 2.85 and 7.75 km, respectively. Eight bats returned to the study roost every night (bats #1–3, #6, #9, #11, #13–14). Of the six remaining bats, four went to a nearby *P. lylei* roost in Prey Veng Province (28 km east, Wat Veal Lbang, Prey Veng, 700 flying foxes), whereas two went to more distant and previously unknown roosting sites: 65 km in one night (site A) and 105 km over two nights (site B) for bat #8 and 50 km during one night (site C) for bat #10 (Figure 4).

### 3.4 Spatial analysis

**Figure 5:**
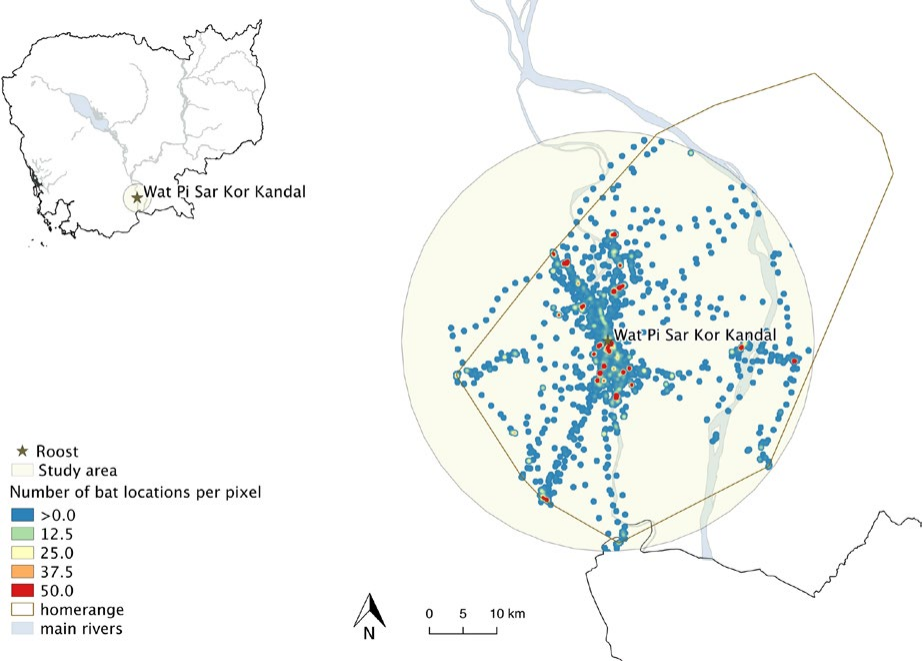
Heatmap of *Pteropus lylei* movements and home range (minimum convex polygon) in southern Cambodia

**Figure 6:**
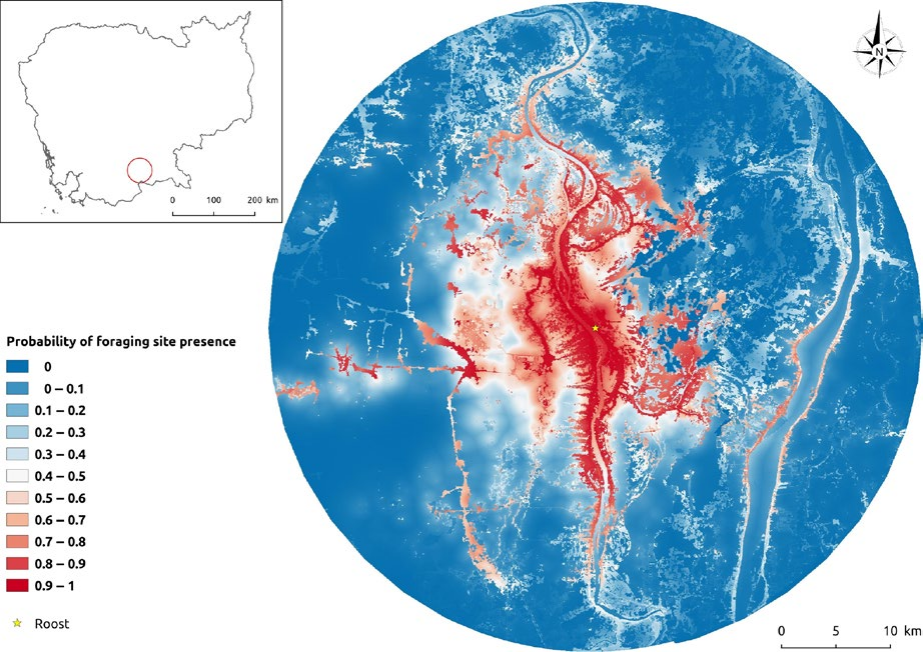
Probability of occurrence of *Pteropus lylei* foraging sites based on spatial distribution modeling, southern Cambodia. The model was trained and validated with GPS locations from 14 tracked individuals

The complete results of the home range estimations for all bats are shown in (Supporting information Table S2). The estimated home ranges were maximal for bats #08 and #10 which went to distant roosts, with 95% home range of respectively 5,984 and 1,158 km^2^. For the eight bats that did not join another roost, the 95% home range ranged from 29.5 to 316.8 km^2^ with an average 95% home range of 104.5 km^2^ (*SD* = 115.5 km^2^). The 50% home range of these same eight bats ranged from 4.3 to 41.1 km^2^ with an average 95% home range of 14.9 km^2^ (*SD* = 13.4 km^2^). Our heatmap of GPS locations showed that most foraging sites and night roosts were located <15 km from the roost (Figure 5). The spatial distribution model showed that foraging locations were significantly negatively correlated with the distance to the roost, residential areas, and water bodies. Conversely, foraging locations were significantly and positively correlated with distance to plantations. Residential areas, trees, and plantations were the main foraging habitats used by the bats (Table 4). Our map of the probability of *P. lylei* foraging sites highlights areas close to the roost but also helps to identify further areas where bat–human interfaces could occur (Figure 6). Model performance was very good with a cross‐validated AUC of 0.93.

**Table 4:** Results of generalized linear model. Significant explanatory variables with a *p*‐value <10^−3^ are given in bold

Variable	Coefficient (*SE*)	*p*‐Value
**Intercept**	2.844 (0.355)	1.10 10^−15^
**Habitat type**		
**Residential area**	**2.853 (0.385)**	**1.34 ** **10^−13^**
**Tree vegetation**	**2.178 (0.296)**	**1.77 ** **10^−13^**
**Plantation**	**1.865 (0.519)**	**3.26 ** ** 10^−4^**
Bare soil	0.695 (0.345)	0.044
Water	0.289 (0.670)	0.666
Flooded vegetation	−0.598 (0.499)	0.231
Shrubland	−13.879 (486.4)	0.977
Rice field	Reference	
**dResid**	**−0.337 (0.111)**	**2.28** ** 10^−3^**
dTree	−0.519 (0.411)	0.206
**dWater**	**−0.599 (0.135)**	**9.38 ** **10^−6^**
**dPlant**	**0.133 (0.040)**	**8.91 ** **10^−4^**
**dRoost**	**−0.220 (0.016)**	**<2 10^−16^**

## 4 DISCUSSION

Our study yielded two main results. First, our study bats mostly foraged in residential areas (53.7% of foraging locations) rather than in plantations (25.6%) and our spatial model indicated that residential areas were the preferred foraging habitat (Table 4). While other studies have shown that *P. lylei and P. giganteus *can primarily forage in anthropogenic landscapes (Hahn et al., 2014; Luskin, 2010; Weber et al., 2015), our data indicate a particularly strong interface through residential backyards where the potential for contact between bats and humans would be higher due to continuous human presence. This could potentially facilitate NiV transmission to humans and domestic animals and two transmission routes have been documented in previous outbreaks of NiV. The first is directly from bats to humans due to consumption of raw palm sap contaminated by flying foxes, which has led to recurrent outbreaks in Bangladesh (Luby et al., 2009). The second route was suggested for the Malaysian outbreak where pigs were likely infected after consuming fruit contaminated by flying foxes (Chua, 2003) and supported by isolation of the virus from fruit partially eaten by bats in Malaysia (Chua et al., 2002). Consistent with this second route, a direct bat‐to‐human transmission via ingestion of fruit has been suggested for another paramyxovirus in Malaysia (Yaiw et al., 2007). Thus, by frequently foraging in residential areas, *P. lylei* could contaminate fruit where humans and domestic animals live, increasing the chance of indirect contact. As such, further information on the use by local residents of fruit partially eaten by bats would help to characterize transmission risks and inform preventative actions including promotion of public awareness. Similarly, palm sap collectors in the study area reported seeing flying foxes on palm trees and urine and feces on collection containers. As our data also indicate that *P. lylei* visits these trees (Table 2), research on palm sap collection in the area is needed to assess the risk associated with this potential transmission route.

Our finding that *P. lylei* mostly forages in residential areas—which mostly correspond to backyards—rather than in plantations was unexpected because human disturbance would likely be higher in the former and food availability greater in the latter. Since our data indicate that *P. lylei* feeds on a variety of fruit in April–May, the greater diversity of fruit typically found in backyards compared to plantations could possibly explain this. More generally, the link between flying fox foraging behavior and the greater diversity of fruits in anthropogenic versus natural environments has been reported elsewhere (Hahn et al., 2014; Luskin, 2010; Weber et al., 2015). All foraging sites in our study were located in anthropogenic landscapes and all individuals showed fidelity to foraging areas, indicating repeated utilization once a food resource was located. This is presumably more energy‐efficient than random foraging and is consistent with studies of *A. jubatus* in the Philippines (de Jong et al., 2013) and *P. alecto *in Australia (Palmer & Woinarski, 1999). From an epidemiological standpoint, an infectious flying fox repeatedly shedding virus in the same area could facilitate site contamination and increase the risk of transmission to humans or animals. Indeed, all of our 14 bats shared at least one foraging site during the study. Repeated shedding at a shared foraging site or night roost could also increase pathogen transmission in the bat population through fruit contamination. In future analyses, we will use a hidden Markov model to determine different phases of nightly movements and attempt to differentiate foraging sites from night roosts.

From a conservation perspective, the apparent preference for backyards and plantations suggest that our *P. lylei* population is highly dependent on human activities for foraging. As such, understanding of community knowledge, attitudes, and practices regarding bats will be important to develop appropriate conservation and public awareness strategies and is now underway. Nevertheless, that residential backyards were the most strongly selected foraging habitat suggests that conflict with humans may be limited in our study area. This is consistent with the fact that other patches of trees were also attractive to our study bats (“Tree vegetation” in Table 4), albeit less than backyards and plantations. Were major bat–human conflicts to occur in our study area, the few attractive non‐human‐dominated habitats present could possibly become overselected by the bats. However, our results must of course be interpreted with caution as only 14 bats in the same population were studied.

Second, because six of our 14 study bats visited at least one other roost during our 28‐day study, it would appear that movements to other roost sites are relatively frequent. However, these movements were limited in time and the fidelity shown to the day roost by all of our study bats is consistent with the non‐nomadic ecology attributed to *P. lylei*. Similar to observations for *P. vampyrus* (Epstein et al., 2009) and *P. medius* (Epstein, unpublished), visits to four other roosts including one 105 km from the study site were observed. These frequent exchanges between roosts are consistent with a regional circulation of different NiV strains in Southeast Asia suggested in previous studies (Epstein, 2017; Wacharapluesadee et al., 2016). From a conservation perspective, they also suggest that *P. lylei* in Cambodia is likely a metapopulation and that conservation strategies should be planned on a regional scale. This is consistent with the results of another telemetry study on the migratory *P. vampyrus, *calling for a comprehensive protection by regional management plans across their international range (Epstein et al., 2009).

The main limitation of our research is the small number of individuals we could study. With only 14 nonrandomly selected individuals tracked out of an estimated 4,000–6,000, our data are unlikely to be representative of the roost population as a whole. Additionally, because foraging behavior is highly dependent on local environments, our results should not be extrapolated to all *P. lylei* colonies in Cambodia. Furthermore, our study group had a strong male bias, with only one female tagged with the GPS device. Though other females were caught, these were excluded as they were pregnant or lactating and because limited data are available for female *P. lylei*, it remains unclear if the sexes differ in their foraging behavior. For instance, while female and male *P. poliocephalus* are similar in their movement patterns (Roberts et al., 2012; Tidemann & Nelson, 2004), lactating females of *P. alecto* travel greater distances between roosts and foraging sites than males (Palmer & Woinarski, 1999; Roberts et al., 2012). Nine of the 14 GPS collars we deployed lasted for at least 10 nights (average 11.8 nights), and 80% of the data were valid. Three other collars provided relatively few valid locations, and only one failed to transmit meaningful data. This performance rate was probably influenced by extended battery life due to high temperatures during the study period, while the open agricultural landscape of our study area probably facilitated the acquisition of GPS locations, saving further battery power. We deployed GPS devices on a limited number of individuals, preventing us from any generalization of the observed patterns at the population level. However, the results were consistent between the different individuals and provided useful information on the movement and foraging ecology of *P. lylei* in Cambodia. The GPS devices we used were battery‐powered, and the size of the battery was limited by the body weight of the flying foxes. By programing five GPS devices to record locations every 30 min instead of 5 min for the nine other devices, we expected them to last for a month. However, data for only two of these bats were collected for more than 20 days, limiting our capacity to observe any change in foraging patterns over this period. Further studies should then be implemented to assess any variability of foraging patterns over time.

While our data represent a brief snapshot in time, they nonetheless illustrate the potential for foraging behavior to potentially facilitate NiV transmission to humans and domestic animals. To date, no transmission from *P. lylei* to human or animals has been recorded despite the circulation of NiV in this species in Cambodia and Thailand (Cappelle et al., 2014; Reynes et al., 2005; Wacharapluesadee et al., 2010). The presence of a hazard such as the NiV in a reservoir population does not necessarily lead to an emergence (Hosseini et al., 2017). Indeed, despite NiV being detected in *P. hypomelanus* on Tioman Island, no outbreak has occurred there, and no evidence of the virus has been found in people on the island (Chong et al., 2003). As such, close and frequent interfaces between bats and humans, including bats roosting in the middle of villages and feeding on cultivated fruit in residential backyards and orchards (Aziz, Clements, Giam et al., 2017) may not be sufficient to lead to an emergence. Other factors such as cultural and agricultural practices must be taken into account.

Different agricultural practices may lead to different levels of exposure in the countries of Southeast and South Asia. Conditions specific to intensive pig farming in Malaysia or palm sap collection in Bangladesh may explain why the virus emerged in these countries. Nevertheless, understanding the ecology of *P. lylei* may significantly improve our ability to target limited resources for interventions, and educational campaigns that discuss the risks of NiV to people and their domestic animals (Nahar et al., 2014; Parveen et al., 2016). In particular, while based on only 14 individuals, our mapping of the probability of occurrence of foraging sites for the *P. lylei* will help targeting prevention measures to areas where contact between flying foxes and humans can be expected.

## CONFLICT OF INTEREST

None declared.

## AUTHOR CONTRIBUTIONS

JC and AT conceived the study and designed methodology; JC and TH coordinated the capture of the bats with the help of KC, SR, NF, VH, and CN; JHE coordinated the deployment of the GPS collars; AT, MG, and AJ collected environmental data and produced the land cover map of the study area; KC and SR collected the GPS data from the collars; KC and SR analyzed the data and led the writing of the manuscript. KC, SR, and JC drafted the first version of the manuscript and all authors contributed critically to the drafts and gave final approval for publication.

## Data Availability

The data used in this study are available on Movebank (movebank.org, study name "Foraging movements of Lyle's flying foxes in Cambodia (data from Choden et al. 2019)") and are published in the Movebank Data Repository (Choden et al., 2019). https://doi.org/10.5441/001/1.j25661td.
