# Supplementary material for: Pteropus lylei primarily forages in residential areas in Kandal, Cambodia
Source: Ecol Evol. 2019 Mar 13;9(7):4181–91. doi: 10.1002/ece3.5046 (PMC6468066; doi:10.1002/ece3.5046)

**Lyle’s Flying Fox, *Pteropus lylei* primarily forages in residential areas in Cambodia**

**K. Choden*, S. Ravon*, Jonathan H. Epstein, T. Hoem, N. Furey, M. Gely, A. Jolivot, V. Hul, C. Neung, A. Tran and J. Cappelle**

**Supplementary Information: Landsat8 image classification**

**Satellite images**

We acquired a cloud-free high spatial resolution Landsat8 image covering the study area (Acquisition date: 2015/04/21). Characteristics of the Landsat scene are presented in Table S1.

**Table S1: Characteristics of Landsat8**

| Band number | Wavelength (nm) | Spatial resolution (m) |
| --- | --- | --- |
| 1* | 433-453 | 30 |
| 2* | 450-515 | 30 |
| 3* | 525-600 | 30 |
| 4* | 630-680 | 30 |
| 5* | 845-885 | 30 |
| 6* | 1560-1660 | 30 |
| 7* | 2100-2300 | 30 |
| 8** | 500-680 | 15 |
| 9* | 1360-1390 | 30 |
| 10*** | 10600-11200 | 100 |
| 11*** | 11500-12500 | 100 |

*: visible-near and short wavelength infrared bands; ** panchromatic band; *** thermal infrared

**Pre-processing**

Pre-processing steps included radiometric calibration and resampling of the multispectral images to the higher resolution of the panchromatic one (15 m). Also texture indices (homogeneity indices computed from the grey level co-occurrence matrix) (Haralick et al., 1964) were derived from the panchromatic band. The accuracy of the spatial registration was verified on the field.

**Classification**

A supervised classification using the maximum likelihood classification algorithm was carried out (ENVI 5.1. software). This method requires field knowledge to define the different classes. A field mission was organized in May 2015 in the study area and eight main land cover classes were identified: residential areas, free water, rice fields, plantations, trees, shrubland, flooded vegetation, and bare soil. For each class, 40 training areas of known land cover (sites identified and georeferenced in the field using Trimble Yuma rugged tablet computer in May 2015) were used. The bands and indices to be included in the classification process were selected from a signature separability test (Jeffries-Matusita distance).The maximum likelihood classification was performed on the reflectance values of the 6 multispectral bands of Landsat8 image (bands 2: blue, 3: green, 4: red, 5: near infrared, 6 and 7: short wavelength infrared) and the two texture indices (homogeneity calculated with 51x51 and 15x15 window sizes).

**Post-classification**

Some post-classification operations (merging, splitting) were required to produce the final land cover. In particular, ‘tree’ pixels adjacent to ‘plantations’ were re-classified as ‘plantation’. On the other hand, plantation areas with a surface inferior to 1 ha were re-classified as ‘trees’. Isolated ‘residential areas’ pixels were reclassified as ‘bare soils’.

**Validation**

The accuracy of the classification was assessed using a new set of training data (ground control points that were not used for the classification). For each class, 20 validation areas were used. In the error matrix, the allocated land-cover class of the training sites was compared to the observed land-cover class and the quality of the classification was measured through the overall accuracy coefficient and the Kappa index (Congalton, 1991). The overall accuracy measures the proportion of ground truth pixels that were correctly classified. The Kappa index represents the proportion of agreement obtained after removing the proportion of agreement that could be expected to occur by chance. It ranges from 0 (poor agreement) to 1 (perfect agreement).

**Results**

The resulting land cover map is presented in Supplementary Fig.1 . Supplementary Fig. S2 shows this same land cover map with the location of *P. lylei* foraging sites captured from GPS data.

Accuracy measures of the land cover map showed a very good agreement between predicted and observed classes with a global accuracy rate of 93.5% and a Kappa index of 0.92.

**References**

Congalton R: A review of assessing the accuracy of classifications of remotely sensed data. Rentote Sensing of Environ 1991, 37:35–46.

Haralick RM, Shanmugam K, Dinstein IH: Textural features for image classification. IEEE Trans Syst Man Cybern 1964, 2:610–621.

**Supplementary Figure 1. Land cover map derived from Landsat imagery**


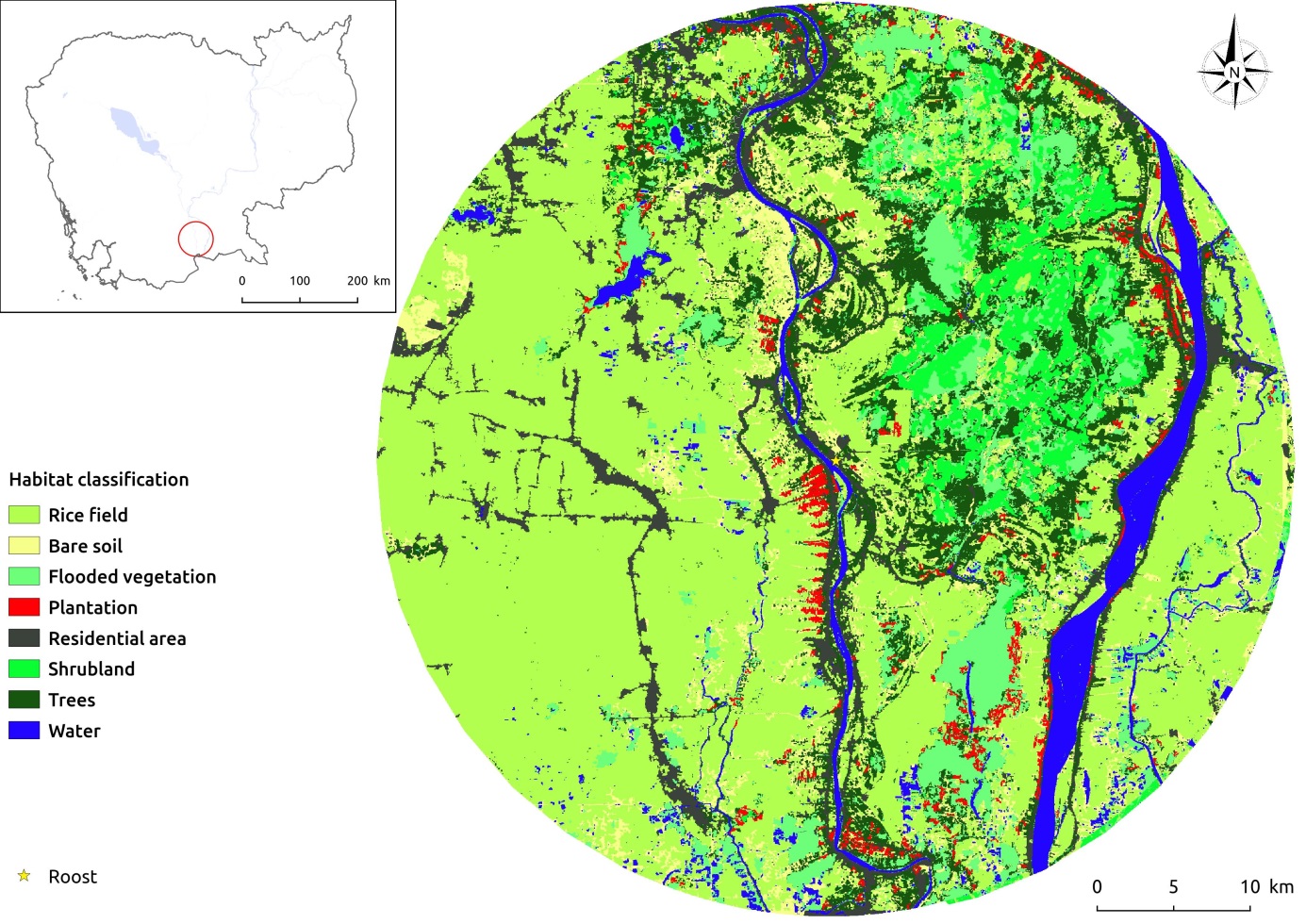


**Supplementary Figure 2. Location of *P. lylei* foraging sites captured from GPS data**


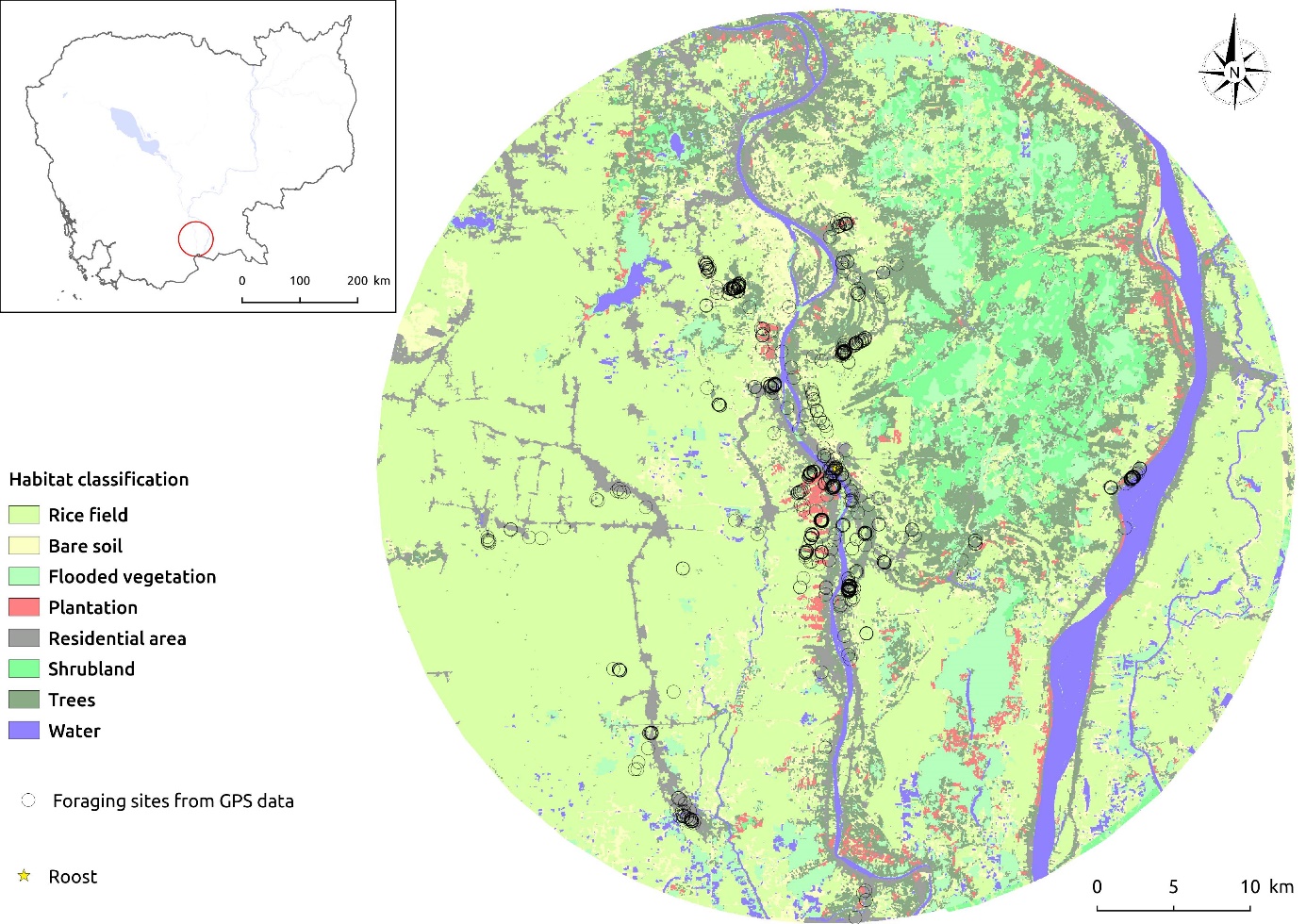

Supplement: Supplementary file 1 [file ECE3-9-4181-s001.docx]
